# A Curious Case of the Persistent Body Stuffer

**DOI:** 10.1155/2019/3948054

**Published:** 2019-09-16

**Authors:** Muhammad Durrani, Carla Dugas, Samaresh Dasgupta

**Affiliations:** Inspira Medical Center, Department of Emergency Medicine, Vineland, NJ, USA

## Abstract

A 29-year-old male presented to our emergency department with complaint of abdominal pain after allegedly ingesting a 4-gram packet of heroin in an attempt to evade detection. Initial evaluation including computed tomography (CT) of the abdomen/pelvis with intravenous and oral contrast, as well as laboratory workup was negative and the patient was discharged. The patient returned 3 days later with complaint of “I feel high” and severe constipation, and demonstrated an opiate toxidrome requiring naloxone with improvement of symptoms. A repeat CT of the abdomen/pelvis, this time without contrast revealed a 2.1 × 1.8 cm foreign body in the gastric antrum. The patient was promptly taken to endoscopy with surgical backup. Foreign body removal included multiple plastic bags encasing heroin, which had sustained a small leak causing a gastric outlet obstruction as well as a slow opiate toxidrome. The foreign body was removed and the patient was observed and discharged with a favorable outcome.

## 1. Introduction

Body packers and body stuffers utilize intracorporeal concealment of illicit drugs in individuals with the aim of eluding police and other agencies and avoiding detection [1–3]. These two subsets of individuals are frequently brought to the emergency department (ED) for either acute drug ingestion, symptoms of intestinal obstruction, or for medical clearance [4–6]. Differentiating between body packers and body stuffers is based on the reason for concealment, amount of drug ingested, and the method of concealment [1, 7, 8]. Body packers, often referred to as “mules”, “couriers”, and “swallowers” are individuals who intentionally and methodically “swallow or insert drugs into a body cavity with the purpose of smuggling them across secure borders” [4]. A “body stuffer” on the other hand is typically a drug user who quickly swallows and inserts the drugs to evade detection without the benefit of time to allow for careful thought and preparation of the drug packages prior to ingestion. “Body packers usually carry about 1 kg of drug, divided into 50 to 100 packets” which vary in size and construction and are “extremely well crafted with a sophistication that suggests an automated process” [4, 9, 10]. To slow down intestinal transition time, “constipating agents and parasympathetic agents are often used to prevent the passage of packets” [11]. Upon arrival, the use of laxatives and other voiding agents are used to accelerate the passage of packets [11]. Body packers thus tend to ingest larger quantities of high purity drugs in securely wrapped, durable packaging, whereas body stuffers typically ingest smaller quantity, less pure, and inadequately wrapped drugs quickly in an attempt to evade imminent detection [7, 12]. It should be noted that in both body packers and stuffers, each packet typically contains a life threatening dose of drug. Hence, body stuffers pose a particularly increased threat of “liberating substantial amounts of drug secondary to the unplanned ingestion with inadequate packaging, which was never intended for gastrointestinal (GI) transit, with devastating consequences and reported fatalities” [7].

The most frequent cause of death among body packers and stuffers is the rupture of ingested drug packets causing an overwhelming acute toxidrome in addition to complications resulting from the size and number of packet ingestions [5, 11]. Ingesting large or numerous foreign bodies and slowing down GI transit can cause intestinal obstructions, which can lead to bowel perforation and death [11]. In addition, complications arising from packet rupture can be unpredictable and diverse, due to the alteration of drugs with other substances, which presents a diagnostic and therapeutic challenge for the emergency medicine physician [4, 11]. Given the difficulty in obtaining an accurate history, and the often nonspecific clinical presentation and laboratory tests, radiographic modalities play an important role in the diagnosis, follow-up, and management [1]. For the emergency department physician, abdominal X-ray, ultrasonography, and computed tomography (CT) are the mainstay of imaging, with noncontrast CT considered to be the study of choice [1, 13–15]. Treatment is multifactorial, based on type and amount of ingested drug, and the presence of symptoms. Typically, asymptomatic patients require a conservative approach to package passage while symptomatic patients require urgent treatment, as well as surgical intervention in select cases.

## 2. Case Presentation

A 29-year-old incarcerated male with no past medical history arrived to our emergency department with chief complaint of abdominal pain. The pain started 3 days prior, after ingesting a packet filled with 4-grams of heroin. The abdominal pain was diffusely localized with no alleviating or exacerbating factors. The pain was described as an achy, constant sensation and was noted to be mild in terms of severity with no other associated symptoms. Upon further history taking from the prison guards at bedside, it was noted that 3 days ago, the patient had been observed quickly swallowing an unknown object given to him by a visitor during visitation hours. He was immediately placed in a solitary confinement unit. Per prison policy, a guard was assigned to inspect the inmate's stool in search of the unknown ingested object. Just prior to presentation to the ED, several small pieces of what they believed to be either a rubber balloon or plastic bags were observed in his last bowel movement. When they mentioned this to the patient, he became concerned that the package had ruptured, and he was brought to the ED for evaluation.

Upon arrival, his blood pressure was 126/76 mmHg, pulse 90 beats per minute, respiratory rate 17 breaths per minute, pulse oximetry 100% on room air, and temperature of 98.0 Fahrenheit. His blood glucose was 109 mg/dl. His electrocardiogram revealed normal sinus rhythm with a rate of 93 beats per minute with no noted abnormalities. Lab work obtained included a complete blood count, complete metabolic panel, urinalysis, as well as a urine drug screen (UDS), which were found to be unremarkable. The patient had a similarly unremarkable physical examination. An obstruction series radiograph with chest radiographs were obtained showing no evidence of free air or intestinal obstruction, and no evidence of foreign body in the esophagus, lungs, and abdomen. At this time, a computed tomography (CT) of the abdomen and pelvis with intravenous and oral contrast was obtained. The CT revealed no acute abdominal pathology and no foreign body. The patient continued to insist that he had swallowed a heroin filled balloon and felt that it may have ruptured. The case was discussed with the prison physician, and it was deemed that with a negative CT scan, normal vital signs and physical examination, with no lab abnormalities, that the patient could be discharged to the prison facility, where he was placed back in solitary confinement, with strict return precautions.

**Figure 1:**
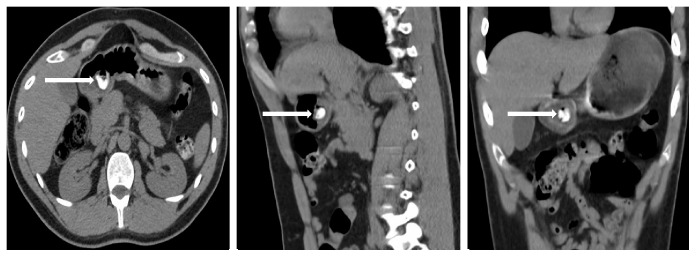
Axial, sagittal, coronal views noting radiopaque density in lumen of gastric antrum (Arrow).

Three days later, the patient returned to the ED claiming that he “felt high” with symptoms of nausea and constipation. He was noted to be have a blood pressure of 151/57 mmHg, pulse 112 beats per minute, respiratory rate 18 breaths per minute, pulse oximetry 95% on room air, and temperature of 98.9 Fahrenheit. His blood glucose was 104 mg/dl. His electrocardiogram revealed sinus tachycardia with a rate of 101 beats per minute and no other noted abnormalities. Repeat workup included a complete blood count, complete metabolic panel, urinalysis, as well as a toxicology workup and repeat urine drug screen (UDS), which were found to be unexceptional aside from positive opiate screen on his UDS. The patient had an unremarkable physical examination. A noncontrast CT of the abdomen and pelvis was obtained revealing a moderately dilated stomach with air fluid levels with a noted radiopaque density in the lumen of the gastric antrum measuring 2.1 × 1.8 cm (Figure 1).

Shortly after the CT scan, the guards alerted the ED staff that the patient was not acting at his baseline mentation. It was noted that the patient was more somnolent with a noted decrease in his respiratory rate to 10 breaths per minute. At this time, the patient received naloxone 0.4 mg intravenously with noted immediate improvement in his mentation and sensorium. His repeat physical examination revealed no neurologic deficits. With findings of a foreign body on the CT scan with a corresponding acute opiate intoxication, emergent gastroenterology and surgical consult for presumed rupture of opiate filled balloon in the gastric antrum was obtained.

**Figure 2:**
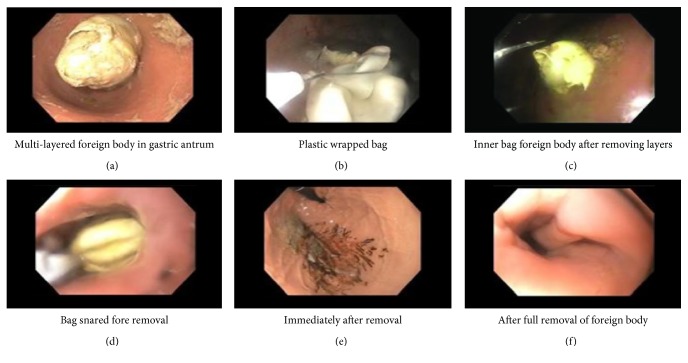
Endoscopic removal of gastric antrum foreign body.

The gastroenterologist proceeded to endoscopy to remove the ingested foreign body with surgical back up present in the operating room. The patient underwent endoscopy to remove the foreign body in the antrum of the stomach. The foreign body was then identified as a plastic bag encasing a separate 2 cm plastic bag with heroin inside, which had sustained a microscopic tear causing a slow opiate toxidrome as well as a gastric outlet obstruction (Figure 2).

The heroin bag was removed completely and the patient was monitored in the intensive care unit for heroin overdose. Prior to discharge, the patient was able to have a normal bowel movement and had resolution of his symptoms. The patient was discharged to prison with a diagnosis of gastric outlet obstruction secondary to foreign body in setting of body stuffing with a hastily prepared packaging of heroin.

## 3. Discussion

**Figure 3:**
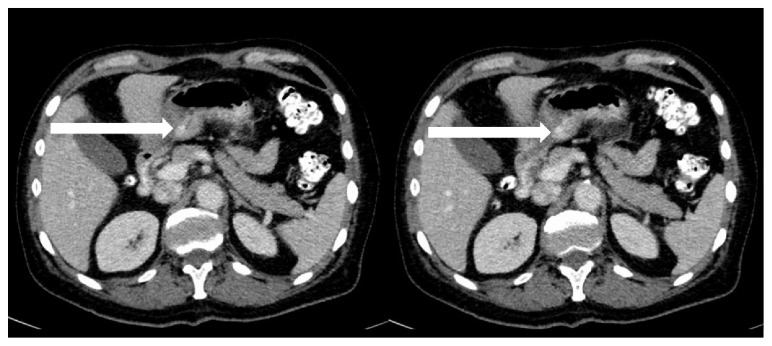
Axial views with oral and IV contrast demonstrating no foreign body in the stomach (Arrow).

Body packing and body stuffing are unique clinical entities that often present both a diagnostic and therapeutic dilemma for the emergency medicine physician due to a number of potential ingestions and coingestions and the often unreliable or unwilling history on the part of the patient. Although relatively rare, there is an increasing trend in this phenomena since the first description of body packing in 1973 by Deitel and Syed [16]. It has been hypothesized that this may stem from “increased border safety measures put in place after the events of September 11, 2001 which made conventional trafficking of illicit drugs more difficult, as well as increased airport security, in addition to steady increases in the global drug trade” [4, 17, 18]. Hence, although uncommon, it is important to be able to identify, diagnose, and treat the life threatening complications that can result from this practice. The diagnosis should include a thorough history which aims to identify “substance ingested, number of packet ingestions, and the nature of the packaging, in addition to the symptoms experienced, paying particularly close attention to gastrointestinal symptoms” [4]. As previously mentioned, the history obtained is frequently unreliable secondary to an unwilling or dishonest historian given the potential ethical, and legal ramifications. Similarly, a thorough physical examination is essential to identify drug toxidromes but is often difficult given the potential for multiple drug ingestions or asymptomatic patients. In the case presented, the patient's second visit to the ED revealed an opiate toxidrome manifesting as decreased respiratory drive, somnolence, constipation, and nausea, requiring naloxone in setting of a microscopic rupture of the ingested heroin packets. It is hypothesized that given the negative UDS and asymptomatic presentation 3 days prior that the patient had sustained a microscopic leak from the ingested heroin packet, which led to his subsequent symptoms and clinical course. It should be noted that since the packets tend to leak before they rupture, “signs and symptoms related to a specific drug should be aggressively pursued early in the assessment to identify impending catastrophic effects” [4]. A focused physical examination that encompasses the patient's vital signs, mentation, pupil size, bowel sounds, and skin findings in addition to an abdominal examination and potential rectal and/or vaginal examination is recommended [4]. As clinical history, examination and laboratory tests frequently fail to make the correct diagnosis, imaging modalities have assumed a central role in the diagnosis of these individuals. Although plain abdominal radiography is often the most widely used and initial modality employed to detect these drug-filled foreign bodies, studies have shown a wide range of sensitivity ranging from 40 to 100% [1, 2, 11, 19]. Despite this large range of sensitivities, most clinicians agree that the utility and sensitivity of plain abdominal radiography tends to be lower end and does not approach 100%. The current practice of radiographically identifying these individuals is transitioning to the use of CT scans, especially in the setting of negative abdominal radiographs. This change in practice and the transition to CT scans is due in part to the drastic reduction in radiation dose with newer generation CT scanners, with sensitivity ranging from 95.6 to 100% [14, 15, 20]. Of note, the current radiology guidelines recommend the use of noncontrast CT scans to adequately identify drug-filled packets as contrast material may cloak ingested packets due to similarities in their density [15]. Here, we entertain 2 theories as to why this patient had an entirely negative workup on his first visit to the ED. The first is that the use of IV and particularly oral contrast in the first CT obscured the foreign body due to similar densities. The second is that the CT scanner missed the foreign body entirely, and it had not yet obstructed the gastric antrum, although this is highly unlikely given that it was 2 cm in diameter. We believe it was likely that the use of oral contrast in the first CT scan led to intraluminal contrast media obscuring the foreign body, although there may have been one present at the time (Figure 3).

Although rare, there have been case studies that have shown than a foreign body may be obscured by intraluminal contrast media leading to false negative CT scans. Ultimately, this patient was appropriately managed with emergent endoscopy and close observation for continued opiate toxidrome and the patient was discharged with a favorable outcome.
